# Quantification of the Proportion of Unfavorable Clinical Outcomes among Imported Malaria Patients According to the Degree of Semi-Immunity on Population Level: An Ecological Study

**DOI:** 10.4269/ajtmh.21-0196

**Published:** 2021-06-14

**Authors:** Tamara Nordmann, Saskia Dede Davi, Michael Ramharter, Johannes Mischlinger

**Affiliations:** 1 Department of Tropical Medicine, Bernhard Nocht Institute for Tropical Medicine & I. Department of Medicine University Medical Center Hamburg-Eppendorf, Hamburg, Germany;; 2 German Centre for Infection Research (DZIF), Hamburg-Luebeck-Borstel, Hamburg, Germany

## Abstract

The protective effect of semi-immunity to alleviate clinical complications of malaria remains incompletely understood. This ecological study quantified the proportion of unfavorable clinical outcomes among patient populations with imported malaria as a function of the reported proportion of absent semi-immunity in a patient population. Group-level proportions were extracted from published studies on imported malaria. Linear regression analyses demonstrate a consistent positive trend between the average proportion of absent semi-immunity in patient populations of imported malaria and the proportion of unfavorable clinical outcomes therein. Regression equations provide a group-level estimate of attributable fractions of clinical complications resulting from absent semi-immunity to malaria.

During the past few years, the proportion of imported malaria attributable to immigrants and travelers visiting friends and relatives has increased consistently.^1^ Patients originating from endemic regions may differ from classical tourists from non-malaria endemic regions because of partially retained semi-immunity, which they acquired during several years of routine exposure to the parasite *Plasmodium falciparum.* As a consequence, fewer clinical complications are noticed among patients with semi-immunity. Despite this well-accepted association, only a limited number of high-validity studies on imported malaria have stratified clinical complications by immunity status. To collate all currently available evidence, we conducted an ecological study in which we quantified the average proportion of unfavorable clinical outcomes among patient populations with imported malaria as function of the reported proportion of absent semi-immunity in a patient population.

For this study, a comprehensive review of the work by Mischlinger et al.^2^ comparing semi-immune and non-immune travelers with imported malaria constituted the sampling frame for the selection of relevant literature. Additional literature was identified in reference lists of respective publications, and group-level data were extracted. The exposure of interest was the proportion of absent semi-immunity in a patient population of a published study on imported malaria. In addition, the proportions of the following unfavorable clinical outcomes were extracted: severe malaria, admission to the intensive care unit (ICU), and death. Linear multivariable regression models were applied to determine the association between absence of semi-immunity and either severe malaria, ICU admission, or death. To prevent heteroskedasticity, weighted linear regression was performed by including the proportion of absent semi-immunity as an analytical weight. Because of the apparent associations with absence of semi-immunity and unfavorable clinical outcome, the following variables were regarded as a priori confounders and therefore data were also collected: age, the proportion of adherence to chemoprophylaxis, and infection by *P. falciparum*.^2,3^ In the univariable analysis, gender was not associated with any outcome. To avoid selection bias, articles were excluded if an outcome served as an inclusion criterion in the original publication.

The proportion of patients with absent semi-immunity in a published article was regarded as the reported proportion of patients without previous exposure to malaria. If a publication did not report explicit information, the proportion of patients who were born and are normally residing in a non-malaria-endemic area was used as the surrogate proportion for the proportion of patients without previous exposure to malaria. The definition of severe malaria followed WHO criteria, unless stated otherwise in the original publication.^4^ Stata v. 16 (StataCorp, College Station, TX) was used for statistical analysis.

Fifty-six articles on imported malaria from 1987 to 2017 were included in the analysis. The majority of articles were published from Europe (82%), six articles (11%) from the United States, and one article (2%) each from Australia and the Middle East. Two articles (4%) covered more than one continent. The median percentage of people without semi-immunity against malaria was 36% (range, 0–88%). The median number of study participants in selected studies was 208 (interquartile range [IQR], 86–545), with a median age of 33 years (IQR, 30–37 years), and the median percentage of males was 66% (IQR, 59–72%).

The median percentage of malaria caused by respective *Plasmodium *spp. was 78.4% (IQR, 66.8–90.3%) for *P. falciparum*, 10.8% (IQR, 0.7–19.4%) for *P. vivax*, 2.4% (IQR, 0–5.2) for *P. ovale*, and 1.2% (IQR, 0–2.3%) for *P. malariae.* The median percentage of participants who reported having taken antimalarial prophylaxis was 21.6% (IQR, 16.0–39.0%). Among the overall study populations, the median proportion was 14.0% (IQR, 6.7–23.3%) for severe malaria, 6.2% (IQR, 4.1–11.8%) for ICU admission, and 0.3% (IQR, 0–1.1%) for a fatal outcome.

Results of univariable linear regression analysis indicated strong evidence for a positive correlation between proportion of absence of semi-immunity and the proportion of severe malaria in a population (*y *= 0.32*x* + 0.02; *P* = 0.002). Similarly, evidence was found in support of a positive correlation between absence of semi-immunity and a fatal outcome on population level (*y *= 0.023*x* + 0; *P* = 0.02). No evidence for such a correlation was detected for ICU admission *(y = *0.06*x* + 0.08; *P* = 0.6) (Table 1). After adjustment for age, *Plasmodium *spp., and self-reported adherence to chemoprophylaxis, all positive correlations increased, as indicated by an increase in all adjusted regression coefficients. The adjusted regression equation for severe malaria is *y* = 0.42*x *+ 0.02 (*P* = 0.001), for ICU admission is *y* = 0.15*x *+ 0.16 (*P* = 0.31), and for a fatal outcome is *y* = 0.045*x* + 0.015 (*P* = 0.004) (Table 2). To assess the role of missing data, univariable regression equations were re-computed restricted to studies that reported all confounders of interest (Table 2). Almost identical univariable and restricted univariable regression coefficients indicate that missing data did not influence the change between crude and adjusted regression coefficients.

**Table 1:** Crude linear regression equations between the proportion of absent semi-immunity and the proportion of unfavorable outcomes in populations with imported malaria

Outcomes	n	Univariable regression equations	*P* value
Severe malaria	38	*y* = 0.32 (0.12 to 0.51)*x* + 0.02 (–0.1 to 0.13)	0.002
ICU admission	18	*y* = 0.06 (–0.18 to 0.31)*x* + 0.08 (–0.07 to 0.23)	0.6
Death	47	*y* = 0.023 (0.004 to 0.042) *x* + 0 (–0.01 to 0.01)	0.02

ICU = intensive care unit. *y* = regression coefficient (95% CI)*x* + constant (95% CI).

**Table 2:** Crude and adjusted linear regression equations between the proportion of absent semi-immunity and the proportion of unfavorable outcomes in populations with imported malaria

Outcome	n	Restricted univariable regression equations*	*P* value	n	Multivariable regression equations	*P* value
Severe malaria	27*	*y* = 0.36 (0.15 to 0.58)*x* + 0 (–0.13 to 0.12)	0.002	27	*y* = 0.42 (0.18 to 0.65)*x* + 0.02 (–0.24 to 0.27)	0.001
ICU admission	12*	*y* = 0.06 (–0.22 to 0.35)*x* + 0.07 (–0.1 to 0.35)	0.64	12	*y* = 0.15 (–0.17 to 0.47)*x* + 0.16 (–0.18 to 0.5)	0.31
Death	28*	*y* = 0.027 (0.002 to 0.056)*x* + 0 (–0.02 to 0.02)	0.06	28	*y* = 0.045 (0.016 to 0.074)*x* + 0.015 (–0.017 to 0.046)	0.004

ICU = intensive care unit. *y* = regression coefficient (95% CI)*x* + constant (95% CI). Adjusted variables in multivariable models: age, proportion of self-reported use of chemoprophylaxis during travel, and proportion of infection with *Plasmodium falciparum* malaria.

* Univariable regression variables were re-computed restricted to studies with available data on all confounders. Similar regression coefficients indicate that missing data did not influence the change between crude and adjusted regression coefficients.

Adjusted linear regression equations suggest that, among populations with 80% of patients without semi-immunity, 36% (*P* = 0.001) would have severe malaria and 5% (*P* = 0.004) would have a fatal outcome. In comparison, among populations with only 20% of patients without semi-immunity, 10% (*P* = 0.001) would have severe malaria and 2% (*P* = 0.004) would have a fatal outcome.

This ecological study on imported malaria demonstrates strong evidence that the proportion of severe malaria and death is greater in populations with a high proportion of patients without semi-immunity than in those with a substantial number of semi-immune individuals. Regression coefficients also indicate such a positive correlation for ICU admission, which is biologically plausible. The lack of statistical evidence related to the ICU model might, therefore, be most likely explained by a relatively low sample size (n = 12). Evidence for the association between absence of semi-immunity and unfavorable clinical outcomes is found in various observational studies.^5,6^ However, to our knowledge, a linear relationship between immunity status against *Plasmodium *spp. and severe malaria and death has hitherto not been demonstrated. Therefore, the presented models constitute a first attempt to quantify the proportions of severe malaria and death as function of the average proportion of absent semi-immunity in populations of patients with imported malaria.

Describing the relationship between semi-immunity and the clinical presentation in malaria patients is complicated by the difficulties to define distinctively semi-immunity status. Semi-immune populations constitute a heterogeneous group with a certain degree of protection against malaria. Semi-immunity generally requires years of residency in areas of high-malaria endemicity to develop clinically relevant protection.^7^ By moving to a non-endemic area, semi-immunity diminishes over time, subjecting individuals to a risk comparable to non-immune individuals after a period of approximately 10 to 15 years, as reported in a study from Sweden.^8^ However, semi-immunity may also wane earlier without booster exposure to the parasite during visits to endemic areas. This indicates that the important group of travelers visiting friends and relatives might retain a certain degree of protective immunity for years to decades of immigration to a non-malaria-endemic country, but the degree of immunity might vary among individuals. On the contrary, absence of semi-immunity to malaria in populations born in non-malaria-endemic countries is rather homogenous. Also, clinical presentation of non-immune patients with imported malaria is less heterogenous than in semi-immune patients.^2,9^ Therefore, it is believed that by choosing absence of semi-immunity as the exposure of interest, the risk of misclassification is minimal.

The protective effect of antimalarial prophylaxis has been demonstrated repeatedly.^10,11^ According to Vliegenthart-Jongbloed et al., there was strong evidence of a protective effect of antimalarial prophylaxis on severe malaria and ICU admission in cases of adequate adherence to the treatment regimen. In our study, multivariable regression models used self-reported information on whether chemoprophylaxis was taken during travel. Because of the nature of self-reported patient outcomes, some residual uncertainty remains regarding the extent of the adequacy of chemoprophylaxis. To decrease the risk of severe complications after *P. falciparum* infection, early diagnosis and prompt treatment are crucial for the management of malaria. Information about the delay in health-seeking behavior among imported malaria patients could not be included into the final model as a result of data sparsity.

For this study we used severe malaria, admission to the ICU, and death as three different unfavorable clinical outcomes for the analysis, because they were reported most commonly in published articles. Although ICU admission and death are specifically defined events, the WHO criteria applied for the definition of severe malaria have been changed repeatedly between 1987 and 2017. Because WHO criteria have been applied equally for semi-immune and non-immune patients, no differential misclassification is expected.

This ecological study provides the first model for quantifying the proportion of severe malaria and death as a function of the proportion of absence of semi-immunity in populations of patients with imported malaria. Findings of this report are potentially important for medical centers where patients with imported malaria are managed. We believe the results of our linear regression models might help clinical decision makers allocate adequate resources for the management of patient populations with imported malaria by estimating the average percentage of severe malaria and death based on the prevalence of absent semi-immunity in their patient population.

Further research is needed to verify such results at the individual patient level. Furthermore, to overcome the difficulties in defining semi-immunity, additional investigations on biomarkers are needed that reflect both the degree of individual exposure to *Plasmodium *spp. as well as the degree of protection from developing clinically apparent disease.
